# Cross-sectional analysis of the reliability and engagement metrics of YouTube videos on semaglutide for weight loss

**DOI:** 10.1097/MD.0000000000049173

**Published:** 2026-06-05

**Authors:** Tuğba Güler Sönmez, İzzet Fidanci

**Affiliations:** a Republic of Turkey Ministry of Health, General Directorate of Public Health, Ankara, Turkey; b Department of Family Medicine, Faculty of Medicine, Hacettepe University, Ankara, Turkey.

**Keywords:** digital literacy, DISCERN, Global Quality Score, health communication, misinformation, obesity, Semaglutide, YouTube

## Abstract

This study aimed to assess the quality and reliability of health information in the 100 most-viewed YouTube videos related to semaglutide for weight loss, as of December 2024. The study also explored the relationship between engagement metrics and content quality, with attention to the prevalence of misinformation. A cross-sectional evaluation was conducted in December 2024. The top 100 English-language YouTube videos retrieved using the search term “semaglutide weight loss” were analyzed. Each video was assessed using 2 validated tools: the Global Quality Score and the Modified DISCERN (quality assessment tool for consumer health information) instrument. Viewer engagement data – including likes, comments, and views – were recorded. Statistical analyses included descriptive statistics and multiple linear regression to examine relationships between engagement metrics and content quality. Videos from academic and healthcare-affiliated sources generally scored higher in quality assessments, while those produced by individual users tended to lack source citations and balanced information. Although certain engagement metrics, such as the number of likes and comments, showed modest associations with higher Global Quality Scores, view count did not consistently predict quality. A notable portion of user-generated videos lacked discussion of semaglutide’s risks and contraindications. The study highlights the variability in quality among semaglutide-related videos on YouTube. Engagement does not necessarily reflect the reliability of content, underscoring the importance of guiding viewers toward credible health sources. Enhancing digital health literacy and promoting greater visibility of evidence-based content may help improve the quality of health information encountered on widely used digital platforms.

## 1. Introduction

In recent years, the internet has emerged as a principal resource for public health information, with video-sharing platforms playing an increasingly dominant role in shaping health-related perceptions and behaviors worldwide. Among these platforms, YouTube stands out as the most widely used, offering over 2 billion active users access to an extensive range of health content. Compared to other social media platforms such as TikTok or Instagram – which are optimized for short-form entertainment – YouTube allows for longer, structured, and potentially more informative health communication. This makes it a particularly relevant medium for evaluating the dissemination and accuracy of medical information.

One subject that has drawn considerable public attention online is semaglutide, a glucagon-like peptide-1 receptor agonist originally developed for the treatment of type 2 diabetes. Following robust clinical evidence indicating its effectiveness in promoting significant weight loss – up to 15% of body weight in some trials – semaglutide has been approved for obesity management and rapidly gained popularity beyond clinical settings.^[1,2]^ This surge in public interest has coincided with a marked increase in user-generated content about semaglutide on platforms such as YouTube.

The growing prevalence of semaglutide-related content has not come without concern. Recent studies have shown that digital platforms are increasingly exploited to disseminate misleading or incomplete health information, particularly in areas involving pharmaceuticals and weight loss.^[3–5]^ Health misinformation can lead to inappropriate self-medication, poor risk-benefit understanding, and diminished trust in medical institutions. While previous studies have investigated the quality of YouTube videos on topics such as bariatric surgery, cancer therapies, and type 2 diabetes,^[6–9]^ research focusing specifically on glucagon-like peptide-1 receptor agonists like semaglutide remains scarce.

Given the rising global use of semaglutide and its rapid diffusion through digital media, it is critical to assess the credibility and quality of the information presented in these highly accessible formats. YouTube’s algorithmic tendency to promote content based on engagement – views, likes, and comments, rather than accuracy – exacerbates the risk of misinformation overshadowing scientific fact.

Therefore, the present study aims to systematically evaluate the quality and reliability of the most-viewed YouTube videos related to semaglutide and weight loss, using standardized scoring instruments such as the Global Quality Score (GQS) and the Modified DISCERN Score. Furthermore, it seeks to determine whether video engagement metrics are predictive of content quality.

We hypothesize that YouTube videos with higher engagement metrics do not necessarily reflect greater scientific accuracy or reliability, and videos uploaded by academic institutions and healthcare professionals exhibit significantly higher informational quality compared with those produced by individual users.

## 2. Materials and methods

### 2.1. Search strategy and video selection

A systematic search was conducted on YouTube on December 15, 2024, using the keyword “semaglutide weight loss.” The default search settings (by relevance) were applied, and the top 100 most-viewed videos were selected for analysis. Only English-language videos were included, reflecting the primary target audience of global English-speaking users. Videos were eligible if they focused on semaglutide in the context of weight loss or obesity treatment, were publicly available at the time of screening, and had a minimum video length of 60 seconds. Exclusion criteria included non-English videos, duplicates, explicit advertisements, or promotional content by commercial entities, and videos with broken or inaccessible links at the time of evaluation.

**Figure 1.**
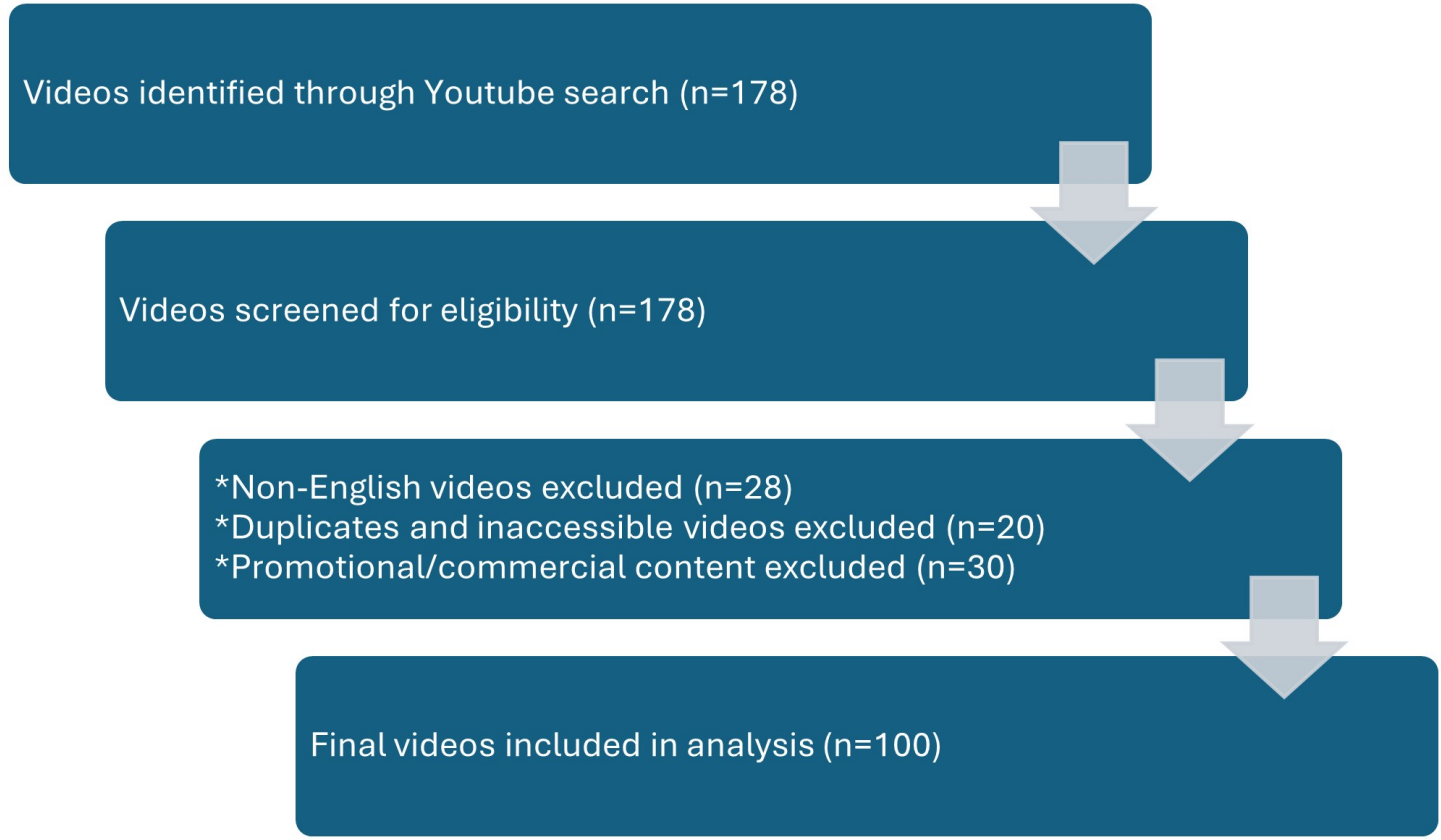
Flow diagram showing the selection of YouTube videos for inclusion in the study, adapted from PRISMA guidelines. PRISMA = Preferred Reporting Items for Systematic Reviews and Meta-Analyses.

A flow diagram illustrating the video selection process is provided in Figure 1. Of the 178 videos initially screened, 100 met all inclusion criteria and were retained for analysis, following exclusions due to duplication, language incompatibility, promotional intent, or inaccessibility.

The sample size of 100 videos was considered adequate and methodologically consistent with previous cross-sectional YouTube content analyses in the medical literature, where the most-viewed videos are commonly selected to reflect the content most likely to influence public perception and engagement.

### 2.2. Data extraction and variables assessed

For each video, the following characteristics were recorded: title, upload date, video duration, total views, number of likes, number of comments, and uploader category (e.g., healthcare professional, academic institution, individual user). In addition, whether the video was sponsored was noted. Viewer comments were not included in the formal content analysis due to their subjective and unstructured nature, although engagement was measured via comment count.

### 2.3. Evaluation tools

Two validated instruments were used to assess video content:

GQS: a 5-point Likert scale used to assess the overall flow, comprehensiveness, and usefulness of the information. A score of 1 indicates poor quality with serious flaws, while 5 indicates excellent quality and high reliability.^[5]^

Modified DISCERN tool: an adapted version of the original DISCERN instrument, evaluating source transparency, citation of evidence, discussion of risks and benefits, and balance of content. Each of the 5 items was scored from 1 (poor) to 5 (excellent), with a maximum total score of 25.^[9]^

Examples were used during training (e.g., videos scoring “1” for GQS lacked scientific structure or used anecdotal claims; videos scoring “5” cited peer-reviewed literature and explained mechanism of action and side effects clearly).

To improve methodological transparency, both evaluation tools were operationalized using predefined scoring criteria. For the GQS, videos were evaluated according to informational flow, clarity, scientific comprehensiveness, and usefulness for patient education. Scores of 1 to 2 indicated poor quality and limited educational value, score 3 indicated moderate quality, and scores of 4 to 5 reflected high-quality, evidence-based, and clinically useful content.

The modified DISCERN instrument evaluated 5 domains: clarity of aims, use of reliable and evidence-based sources, balance and neutrality of information, discussion of risks and adverse effects, and provision of additional sources for patient education. Each domain was scored on a 5-point Likert scale, with higher scores indicating greater reliability and transparency.

Scientific accuracy was assessed by comparing the information presented in the videos with current evidence-based obesity management guidelines, Food and Drug Administration-approved indications for semaglutide, and major clinical trial findings regarding semaglutide efficacy and safety.

Detailed scoring domains and evaluation criteria used for the GQS and modified DISCERN instruments are presented in Table S1, Supplemental Digital Content.

### 2.4. Rater training and reliability

Two independent raters with backgrounds in public health and clinical medicine assessed all videos. Before formal data collection, both raters completed a calibration process using 10 randomly selected pilot videos that were not included in the final analysis. During calibration, raters independently scored each video using the predefined GQS and modified DISCERN criteria, followed by consensus meetings to discuss discrepancies and standardize scoring interpretation.

Examples of low-quality and high-quality videos were reviewed during training. Videos considered low quality typically lacked scientific references, presented anecdotal claims, or omitted adverse effects, whereas high-quality videos cited peer-reviewed literature, discussed mechanisms of action, and addressed safety considerations and contraindications.

The raters evaluated videos according to scientific accuracy, completeness of risk-benefit discussion, transparency of information sources, neutrality of presentation, and patient-oriented educational usefulness. Inter-rater reliability was assessed using the Cohen kappa coefficient, which was calculated as 0.81, indicating substantial agreement between raters.

The use of 2 independent raters, predefined scoring criteria, and calibration sessions was intended to enhance reproducibility and methodological consistency across evaluations.

### 2.5. Blinding and bias mitigation

To minimize potential assessment bias, uploader-related identifiers were masked on the data extraction sheets during the initial quality scoring process whenever feasible. Raters were instructed to focus exclusively on video content characteristics rather than channel popularity or creator identity. In addition, engagement metrics such as views, likes, and comments were recorded separately after completion of quality assessments.

### 2.6. Statistical analysis

Statistical analyses were conducted using IBM SPSS Statistics version 28.0. Descriptive statistics were used to report means and standard deviations for GQS and DISCERN scores. Pearson correlation was used to assess the relationship between video engagement metrics (views, likes, comments) and quality scores. A multiple linear regression analysis was performed to determine whether engagement metrics predicted content quality. The use of regression was justified by the continuous nature of the dependent variable (GQS/DISCERN) and the exploratory aim of identifying linear associations.

### 2.7. Ethical considerations

This study did not require ethical board approval as it involved the analysis of publicly available data without human subjects or personal identifiers. The methodology adhered to ethical principles outlined in prior digital health research literature.

## 3. Results

A total of 100 English-language YouTube videos related to semaglutide and weight loss were included in the analysis. The average video duration was 6.2 minutes (standard deviation [SD] = 3.4), and the majority (58%) were uploaded between 2022 and 2024, reflecting a recent surge in public interest. The most common uploader categories were individual users (38%), healthcare professionals (28%), healthcare institutions (18%), and academic organizations (16%). Among healthcare-affiliated sources, 61% of uploaders identified themselves as physicians or registered medical professionals.

### 3.1. Content characteristics and scientific rigor

While 84% of videos discussed the intended weight loss benefits of semaglutide, only 42% addressed potential side effects (e.g., gastrointestinal symptoms), and 18% mentioned contraindications such as pancreatitis or thyroid C-cell tumors. Videos from individual users often lacked medical references, and 60% of those did not cite any source or included anecdotal claims.

### 3.2. Metrics of engagement and their role as predictors

The rationale for using engagement metrics – views, likes, and comments – was based on YouTube’s algorithmic behavior, which amplifies content that garners more interaction, thereby influencing viewer exposure. Mean values for these metrics were as follows:

Views: 101,250 (SD = 38,500).
Likes: 2350 (SD = 1200).
Comments: 440 (SD = 285).

### 3.3. Quality scores by uploader type

The overall GQS across all videos was 3.4 (SD = 1.2), and the modified DISCERN score was 3.2 (SD = 1.1). Stratification by uploader type is shown in Table 1. Videos from academic institutions received the highest average scores, followed by healthcare institutions and professionals. Individual user videos scored the lowest on both metrics. This table presents the top 100 most-viewed English-language YouTube videos discussing semaglutide, stratified by uploader category. Sample size (n) is indicated for each group.

**Table 1:** Comparison of YouTube videos on semaglutide by source.

Video source	n	Average views	GQS score (mean ± SD)	DISCERN score (mean ± SD)
Academic videos	16	120,000	4.8 ± 0.6	4.7 ± 0.5
Healthcare institutions	18	110,000	4.5 ± 0.8	4.3 ± 0.7
Healthcare professionals	28	95,000	4.2 ± 0.9	4.0 ± 0.8
Individual users	38	80,000	3.0 ± 1.0	3.2 ± 1.1

DISCERN = quality assessment tool for consumer health information, GQS = Global Quality Score, SD = standard deviation.

### 3.4. Regression analysis

Table 2 summarizes the results of a multiple linear regression model evaluating the relationship between engagement metrics (views, likes, comments) and the GQS. Both unstandardized and standardized beta coefficients, along with 95% confidence intervals, are reported. The model was statistically significant, *F*(3, 96) = 5.42, *P* < .01, although the adjusted *R*^2^ = 0.18 suggested moderate explanatory power.

**Table 2:** Multiple linear regression predicting GQS from engagement metrics.

Predictor	Beta (β)	Standard error	95% CI	*P*
View count	0.07	0.05	−0.03 to 0.17	.16
Likes	0.18	0.07	0.04 to 0.32	.02
Comments	0.24	0.08	0.08 to 0.40	.01

CI = confidence interval, GQS = Global Quality Score, β = beta coefficient.

### 3.5. Subgroup analysis by video duration

Videos were stratified into 2 subgroups: short (<5 minutes, n = 41) and long (≥5 minutes, n = 59). Longer videos scored significantly higher on both GQS (3.7 vs 3.1, *P* = .01) and DISCERN (3.5 vs 2.9, *P* = .02). These results suggest that more detailed content may contribute to greater scientific reliability.

A chi-square test confirmed a significant association between uploader type and high-quality content (GQS ≥ 4), χ^2^(3) = 23.9, *P* < .001. Academic and healthcare-affiliated videos were disproportionately more likely to fall in the high-quality category.

**Figure 2.**
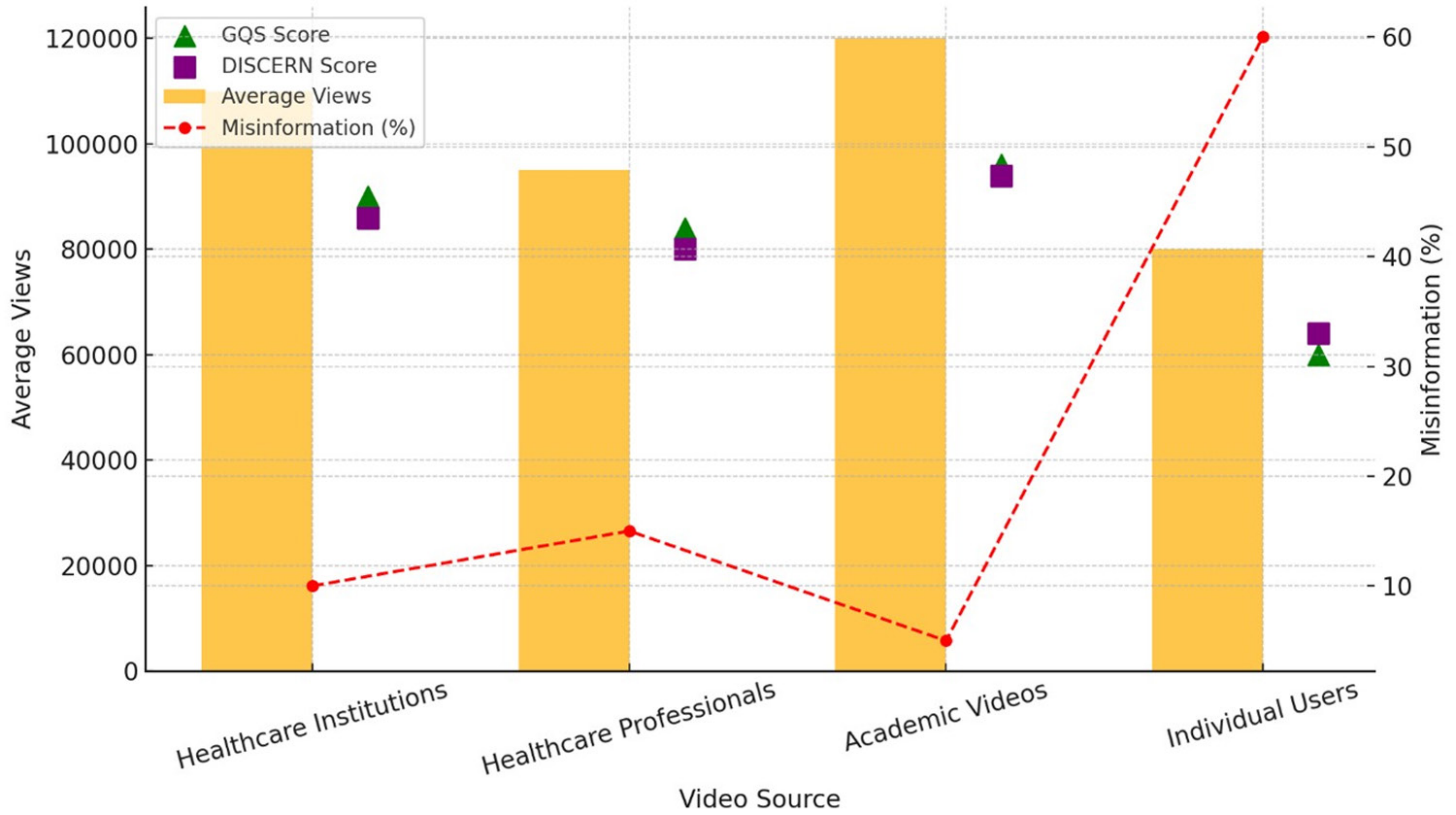
Comparison of semaglutide YouTube content by source. DISCERN = quality assessment tool for consumer health information, GQS = Global Quality Score.

Figure 2 illustrates GQS and DISCERN scores across video sources, highlighting the consistency of higher quality among professionally affiliated content creators.

## 4. Discussion

This study evaluated the quality, reliability, and engagement metrics of the 100 most-viewed YouTube videos discussing semaglutide and weight loss. The average GQS was 3.4 (SD = 1.2), while the modified DISCERN score averaged 3.2 (SD = 1.1), suggesting moderate informational quality across the sample. Videos uploaded by academic institutions achieved the highest GQS (4.8 ± 0.6) and DISCERN (4.7 ± 0.5) scores, whereas content from individual users ranked lowest on both measures (Table 1). Notably, although likes (β = 0.18, *P* = .02) and comments (β = 0.24, *P* = .01) were significant predictors of higher quality scores, the model’s low adjusted *R*^2^ value of 0.18 implies that viewer engagement explains only a small portion of the variance in content quality (Table 2). Therefore, engagement metrics, while statistically significant, should be interpreted cautiously and not equated with informational accuracy. Although the study identified variability in informational quality and completeness, the findings should be interpreted within the context of a cross-sectional content evaluation and should not be considered a direct measure of intentional misinformation.

These results reinforce previous literature emphasizing that content popularity on YouTube does not correspond to scientific credibility.^[4,5]^ The low correlation between views and content quality (*R* = 0.14, *P* = .21) suggests that the platform’s algorithm, which primarily rewards engagement (e.g., watch time, likes, comments), may amplify videos regardless of medical accuracy. YouTube’s recommendation system has been previously shown to contribute to echo chambers and content amplification based on interaction rather than veracity.^[9–16]^

Approximately 60% of the videos produced by nonprofessionals lacked scientific referencing, and 25% overemphasized weight loss while omitting critical information on semaglutide’s risks, such as gastrointestinal side effects and contraindications (e.g., medullary thyroid carcinoma risk). These omissions reflect a broader concern about health misinformation on digital platforms – particularly in pharmacological topics where nuanced benefit-risk discussions are essential.^[3]^

While earlier studies have reported misinformation rates of 40% to 45% in oncology or diabetes-related YouTube videos,^[4,7]^ our analysis of semaglutide-related content reveals a higher proportion of misleading content. However, we caution against overgeneralizing these findings beyond the specific topic and platform. Misinformation dynamics may differ considerably across platforms such as TikTok or Instagram, which have different user demographics, video formats, and moderation systems.^[9]^

Several possible confounding factors could influence both engagement and perceived quality. For example, video production value, thumbnail attractiveness, and speaker charisma may increase viewer interaction independently of content accuracy.^[13]^ These non-content factors were not systematically assessed in this study, representing a potential limitation in interpreting engagement-quality associations.

The findings carry direct implications for clinicians, who may increasingly encounter patients influenced by misleading or oversimplified online content about semaglutide. Physicians and healthcare professionals should be prepared to address misconceptions stemming from social media, using patient education strategies to promote accurate understanding of treatment options, risks, and realistic outcomes.

Moreover, the cross-sectional nature of our research design limits causal inference. As video metrics such as likes and views are dynamic and evolve over time, our analysis provides only a snapshot rather than longitudinal insight. Future studies may benefit from tracking content performance and user feedback over time to assess how misinformation propagates or declines.

From a policy perspective, the findings emphasize the urgent need to incorporate media literacy education into digital health strategies. Empowering users to critically appraise online content – through public campaigns, patient education materials, or school-based interventions – may help counteract the effects of digital misinformation.^[12]^ Furthermore, collaboration with video platforms to adjust ranking algorithms to prioritize evidence-based content could mitigate the spread of misleading videos. Several recent proposals call for hybrid models where verified health organizations partner with tech companies to flag or elevate medically accurate content.^[15]^

Future research should explore automated detection tools to identify low-quality health content, comparative analyses across platforms, and behavioral experiments to assess user trust in videos based on source labeling or quality disclosures. Integrating interdisciplinary methods – combining health communication, data science, and behavioral psychology – will be essential in addressing the growing intersection between social media and public health. Future studies may benefit from the inclusion of experts in health communication to enhance the interpretation of message framing, audience reception, and the persuasive strategies used in video content.

From a public health perspective, the widespread dissemination of unreliable health content on digital platforms represents a growing concern. Policymakers and regulatory bodies should consider developing digital literacy campaigns and platform accountability measures to ensure that evidence-based information is prioritized in algorithmically recommended content.

## Author contributions

**Conceptualization:** Tuğba Güler Sönmez.

**Data curation:** Tuğba Güler Sönmez.

**Formal analysis:** Tuğba Güler Sönmez, İzzet Fidanci.

**Resources:** Tuğba Güler Sönmez.

**Investigation:** İzzet Fidanci.

**Methodology:** İzzet Fidanci.

**Project administration:** İzzet Fidanci.

**Software:** İzzet Fidanci.

**Supervision:** İzzet Fidanci.

**Validation:** İzzet Fidanci.

**Visualization:** İzzet Fidanci.

**Writing – original draft:** Tuğba Güler Sönmez.

**Writing – review & editing:** Tuğba Güler Sönmez.


