# Supplementary material for: Cross-sectional analysis of the reliability and engagement metrics of YouTube videos on semaglutide for weight loss
Source: Medicine (Baltimore). 2026 Jun 5;105(23):e49173. doi: 10.1097/MD.0000000000049173 (PMC13246070; doi:10.1097/MD.0000000000049173)
Supplement: Supplementary file 1 [file medi-105-e49173-s001.docx]

Supplementary Table 1. Scoring Criteria Used for GQS and Modified DISCERN Evaluation

| **Tool** | **Domain** | **Description** | **Score** |
| --- | --- | --- | --- |
| GQS | Flow and clarity | Logical and understandable presentation | 1–5 |
| GQS | Scientific comprehensiveness | Inclusion of evidence-based medical information | 1–5 |
| GQS | Educational usefulness | Practical value for patient education | 1–5 |
| DISCERN | Clarity of aims | Clear purpose and objectives | 1–5 |
| DISCERN | Source reliability | Citation of reliable/scientific sources | 1–5 |
| DISCERN | Balance and neutrality | Balanced discussion without exaggeration | 1–5 |
| DISCERN | Risk discussion | Mention of adverse effects/contraindications | 1–5 |
| DISCERN | Additional resources | Referral to further patient information | 1–5 |
